# Effects of Krill Oil and Race Distance on Serum Choline and Choline Metabolites in Triathletes: A Field Study

**DOI:** 10.3389/fnut.2020.00133

**Published:** 2020-08-18

**Authors:** Andreas B. Storsve, Line Johnsen, Christoffer Nyborg, Jørgen Melau, Jonny Hisdal, Lena Burri

**Affiliations:** ^1^Aker BioMarine Antarctic AS, Lysaker, Norway; ^2^Department of Clinical Medicine, Faculty of Medicine, University of Oslo, Oslo, Norway; ^3^Section of Vascular Investigations, Oslo University Hospital, Oslo, Norway; ^4^Prehospital Division, Vestfold Hospital Trust, Tønsberg, Norway

**Keywords:** choline, betaine, dimethylglycine, high-intensity exercise, krill oil, phosphatidylcholine, sport nutrition, triathlon

## Abstract

Choline is an essential nutrient that has been implicated in athletic performance due to its role in maintaining normal muscle function. The concentration of free choline in serum may decrease during long-distance high-intensity exercise, yet few nutritional strategies to counteract this potentially performance-depleting loss in choline have been investigated outside the laboratory. This exploratory field study was performed to investigate if pre-race supplementation with phosphatidylcholine from krill oil can counteract the expected drop in choline and some of its metabolites during triathlon competitions. Forty-seven triathletes, 12 females and 35 males ranging in age from 25 to 61 years, were recruited from participants in the Ironman-distance Norseman Xtreme triathlon and the Sprint/Olympic-distance Oslo Triathlon. Twenty-four athletes were randomly allocated to the krill oil group, receiving 4 g of Superba*Boost*™ krill oil daily for 5 weeks prior to the race, and 23 athletes were randomly allocated to the placebo group, receiving 4 g of mixed vegetable oil daily. Blood samples were obtained before the race, immediately after completion of the race, and the day after the race for analysis of choline and its metabolites. The results showed that serum choline concentrations significantly decreased from pre-race to race finish in all races, with a more pronounced decrease observed in the Ironman-distance Norseman Xtreme triathlon (34% decrease) relative to the Sprint/Olympic-distance Oslo Triathlon (15% decrease). A reduction in betaine was also observed, while dimethylglycine (DMG) concentrations remained stable across all time points. Significantly higher concentrations of choline (9.4% on average) and DMG (21.4% on average) were observed in the krill oil compared to the placebo group, and the krill oil group showed a significantly greater increase in serum choline following race completion. In conclusion, krill oil may help to prevent that circulating choline concentrations become limiting during endurance competitions.

## Introduction

There has been a marked worldwide increase in the number of people participating in endurance events over the past decade, with considerable variation in distance and level of difficulty across different competition formats. In the case of triathlons, for example, events range from relatively flat 25.75 km sprint- and 51.5 km Olympic-distance races, to Ironman events such as the Norseman Xtreme Triathlon, covering a distance of 226 km, and 5,235 m elevation (1–3). Gaining a better understanding of the unique physiological and nutritional requirements of non-professional athletes across different competition formats will allow for better targeted dietary advice to this group of individuals.

Krill oil is a dietary supplement extracted from Antarctic krill (*Euphausia superba*), rich in the long-chain n-3 polyunsaturated fatty acids (PUFAs) eicosapentaenoic acid (EPA; C20:5 n3) and docosahexaenoic acid (DHA; C22:6 n3) (4, 5). Due to their immunomodulatory and anti-inflammatory properties, EPA, and DHA are associated with benefits for inflammatory-related diseases (6, 7). Given that prolonged periods of high-intensity long-duration exercise may also negatively impact immune function and inflammation, there is considerable interest in marine-based n-3 PUFA supplementation as a means to improve athletic recovery (8). To this end, studies that have investigated the benefits of krill oil in sports settings to date have focused on post-exercise immune function (9), post-exercise oxidative stress (10), and mTOR signaling (11). However, while krill oil is an acknowledged dietary supplement to provide n-3 PUFAs, no sports nutrition studies have looked at the lesser known aspect of krill oil being a rich source of the essential nutrient choline.

Choline is an essential nutrient that has been suggested as a performance-limiting factor in endurance competitions due to its role in maintaining optimal muscle function during prolonged periods of physical activity (12). In contrast to fish oil, where the fatty acids are typically present in triglyceride form, krill oil contains fatty acids that are largely present in phosphatidylcholine (PC) form (13). With a high phospholipid content (≥56%), of which PC makes up around 90%, supplementation with 1 g of Superba*Boost*™ results in a choline dose of ~70–80 mg. A recent single-dose plasma kinetic study in humans showed that krill oil is an adequate source of choline (14). In comparison to choline bitartrate, plasma choline levels were similar, but the metabolites betaine and dimethylglycine (DMG) were significantly higher in the krill oil group, and trimethylamine N-oxide (TMAO) levels significantly lower. Furthermore, another study over 4 weeks confirmed that krill oil treatment significantly increased plasma choline and betaine concentrations in healthy young adults (15). It is not yet known, however, whether supplementation with krill oil will modulate levels of circulating choline and its metabolites in athletic settings where choline depletion is likely to occur.

Choline is essential for the synthesis of neurotransmitters, cell-membrane signaling, transport of fat, and methyl group metabolism (16–18). These cellular functions are the basis for optimal sports performance, but they are challenged during intense physical activity. Inasmuch, plasma choline levels drop during strenuous (above 70% VO_2max_) and prolonged (more than 2 h) exercise, which was shown in endurance athletes such as runners and cyclists (12). If no choline is supplemented, marathon runners can manifest up to a 40% decrease in blood choline levels (19, 20), which was similar in cyclists performing a 2-h cycle ergometer test (21). It can thus be expected that longer distance triathlons, such as Ironman competitions, will result in greater reductions in circulating choline relative to shorter distance triathlons.

It has been suggested that circulating plasma choline is used as a reservoir to cover the increased need for methyl groups during exercise (22, 23). When free choline is not available as a precursor for acetylcholine it can negatively influence muscle performance, since acetylcholine is a neurotransmitter that stimulates muscles to contract (20, 24, 25). If the free choline deficiency state in the blood is persistent, then also PC in cell membranes can be mobilized. However, this increases the risk for compromised cellular membrane integrity and stress resistance (26, 27). Decreased membrane PC and acetylcholine production during strenuous exertion can lead to muscle damage and reduced muscle stimulation, respectively, which might both contribute to the muscle fatigue seen at the end of intense endurance exercise (12).

As only limited amounts of choline can be made by the body itself, the majority of choline needs to be taken up over the diet from e.g., soybeans, eggs, liver, beef, and milk (28–30). Although most diets are considered to provide appropriate amounts of choline, persons at risk of choline deficiency exist. Specifically, the National Health and Nutrition Examination Survey in 2003–2004 concluded that 90% of the American population has an inadequate choline intake (31). The adequate intake levels for choline were set at 550 mg/day for men and 425 mg/day for women in sight of preventing liver damage. A slightly lower adequate intake of daily 400 mg choline for all adults was recommended by the European Food Safety Authority (EFSA), but with 370 mg/day in average choline intake also Europe falls below recommended levels (32). No recommendations for endurance athletes currently exist, but given that physical activity-dependent decreases in circulating choline has previously been reported in the literature, it could be expected that increased intake of choline may help reduce the magnitude of choline depletion, especially in long-duration high-intensity events.

The main objective of the present study was to investigate to what extent circulating, free choline and choline metabolite concentrations are reduced in athletes after completing an Ironman-, Olympic-, or sprint- distance triathlon competition. Further, we aimed to investigate to what extent supplementation with krill oil might be a nutritional strategy to counteract the potential fall in circulating choline concentrations during competitions and restore choline levels faster in the recovery phase. It was hypothesized that (1) the Ironman-distance triathlon would result in a more pronounced drop in serum free choline concentrations relative to the shorter distance triathlons; (2) athletes supplemented with PC-containing krill oil would show higher concentrations of choline and choline metabolites as compared to athletes in the placebo group.

## Materials and Methods

### Participants and Procedures

In this study, non-professional athletes participating in either the Norseman Xtreme Triathlon (in 2018 and 2019), one of the world's toughest triathlons of Ironman-distance (3.8 km swim, 180 km cycling, and 42.2 km run), and the Oslo triathlon (in 2018), which included Olympic distance (1.5 km swim, 40 km cycling, and 10 km run) and sprint distance (0.75 km swim, 23 km cycling, and 5 km run), were recruited via e-mail and randomly allocated into different groups. One group of participants self-administered a daily dose of 4 g Superba*Boost*™ krill oil (Aker BioMarine Antarctic AS, Lysaker, Norway) for 5 weeks prior to race start, and one group self-administered a daily dose of 4 g mixed vegetable oil (provided by Aker BioMarine) during the same 5-week period. The nutritional contents of these supplements are given in Table 1. Briefly, the daily dose of krill oil provided 2,404 mg phospholipids, of which 2,232 mg were PC that contained 316 mg choline. The mixed vegetable oil contained neither phospholipids nor choline. To enter the study, athletes were required to be confirmed participants of the Norseman Xtreme Triathlon or Oslo Triathlon, agree to take and self-monitor use of test products, agree to have blood samples drawn before and after the race, and agree to answer short questionnaires on health, training history, and demographics.

**Table 1:** Characteristics and nutritional composition of study products.

**Product**	**Krill oil**	**Placebo**
Product name	Superba Boost™ krill oil	Mixed vegetable oil
Capsules provided by	Aker biomarine Antarctic AS	Aker biomarine Antarctic AS
Amount per capsule (mg)	1,000	1,000
Capsules per day	4	4
Weeks of administration	5	5
Omega-3 content per capsule (mg; EPA/DHA)	322 (193/96)	4 (<1/ <1)
Phosphatidylcholine content per capsule (mg)	558	<1
Choline content per capsule (mg)	79	<1

Seventy-three individuals agreed to participate in the study and to take supplements at the prescribed dose for 5 weeks prior to race start, and 48 participants were included in the study with a complete set of choline measurements. Of these 48 participants, one participant reported not having taken the study product and was excluded, giving a final sample of 47 (35 males, 12 females) participants. Table 2 presents the characteristics of the subjects included in the analyses. The majority (*n* = 39) of participants took part in the Ironman-distance triathlon, and only eight completed the Olympic and sprint distances. All participants gave written informed consent to participate in the study and ethical approval was obtained from the Regional Committees for Medical and Health Research Ethics in Norway (Protocol number: REK Sør-Øst 2016/932). The study was performed in accordance with the declaration of Helsinki.

**Table 2:** Characteristics of participants by study group (mean ± SD).

	**Krill oil**	**Placebo**	***p***
*N* (male/female)	24 (19/5)	23 (16/7)	
Age (years)	40.1 (10.3)	40.8 (6.8)	0.78
BMI (m/kg^2^)	24.1 (2.0)	23.4 (3.3)	0.35
Exercise (h/week)	13.7 (3.9)	13.3 (5.7)	0.80
Total race time ironman-distance (min)	844 (118)	881 (81)	0.29
Total race time regular-distance (min)	136 (38)	121 (43)	0.64

This study is a sub-analysis taken from a larger study that established biomarkers for athletes participating in extreme endurance competitions (manuscript submitted).

### Blood Sample Collection and Biochemical Analysis

Blood samples were collected by trained medical staff from Oslo University Hospital into vacutainers containing silica particles and gel separators for serum collection. Samples where clotted at room temperature for 30 min before centrifugation at 2,000 g for 10 min and refrigerated. Collection of samples occurred at three separate time points; the first sample was taken <24 h before race start, the second sample immediately after race completion, and the third sample around noon the day after the races.

### Serum Analysis

Serum was used for analysis of free choline and its metabolites at the laboratory of Bevital AS (Bergen, Norway). Measurements were carried out using an 1,100 HPLC system (Agilent Technologies, Santa Clara, United States of America). The HPLC system was coupled to an API3000 triple-quadrupole tandem mass spectrometer (AB Sciex, Framingham, United States of America) equipped with an electrospray ion source and fitted with a hot source-induced desolvation from IONICS (Calamba City, Philippines). Analyst (Ver.1.5.2; AB Sciex, Framingham, United States of America) was used for data acquisition and analysis. Sample processing was performed by a MikroLab AT Plus robotic workstation (Hamilton, Bonaduz, Switzerland) and samples of 10 μL of deproteinized plasma were injected onto a Fortis Phenyl column from Fortis Technologies Ltd (Cheshire, United Kingdom) guarded by a Polar-RP SecurityGuard Cartridge (Phenomenex, Torrance, United States of America). The mass spectrometer was operated in the positive ESI mode, and analytes and internal standards were detected in MRM with unit resolution at quadrupole 1 (Q1) and low resolution at Q3. More details are given elsewhere (33).

Serum concentrations (μmol/L) are reported for the following biomarkers: free choline, homocysteine, cysteine, betaine, Trimethylamine N-oxide (TMAO), trimethyllysine (TML), dimethylglycine (DMG), methionine, creatinine, and arginine.

### Statistics

Repeated measures three-way ANOVA was employed to test for overall and interaction effects of time point (pre-race, 0 h post-race, and 1 d post-race; within subjects factor), supplement type (krill oil and placebo, between subjects factor), and race distance (Ironman-distance and Olympic/sprint-distance, between subjects factor) on levels of free choline and relevant metabolites (μmol/L). Effect size estimates are given as partial eta-square ($\eta_{\text{p}}^{2}$), where 0.01 is considered a small effect size, 0.09 a medium effect size, and 0.25 a large effect size. The assumption of spherecity was tested with Mauchly's test of spherecity, and adjusted *p*-values are given where this assumption is violated. Bonferroni-corrected *t*-tests were employed where appropriate. Alpha was set at 0.05.

## Results

There was no significant difference in choline levels between the Olympic and Sprint triathlon distances across the three time points (*p* = 0.09). Therefore, in order to simplify further analyses, these two races were combined to form a “regular-distance triathlon” group. Furthermore, there were no significant differences in age, sex, BMI, and exercise volume between the krill oil and placebo groups for either race distance (*p* > 0.05).

### Choline

**Figure 1:**
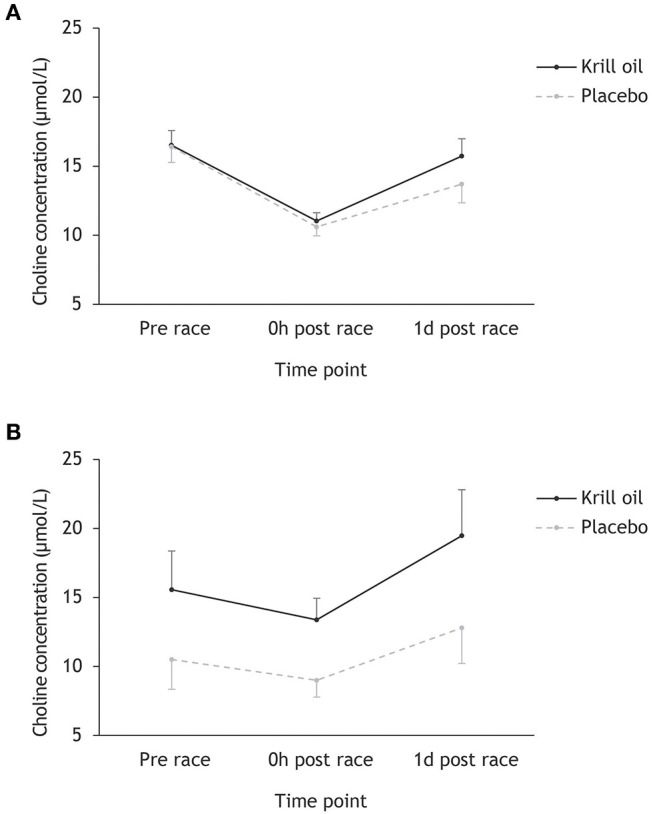
Mean (+/– 95% CI) choline concentrations (μmol/L) for the krill oil group and the placebo group across the three time points. Results are displayed separately for the Ironman-distance triathlon **(A)** and the regular-distance triathlon **(B)**.

Inspection of Figure 1 reveals a decrease in choline levels from pre-race to race finish in Ironman-distance athletes (from 16.5 to 10.9 μmol/L) and regular-distance athletes (from 12.4 to 10.6 μmol/L), with much more pronounced decreases being observed in the Ironman athletes (34 vs. 15% decrease). These levels are largely recovered the day after the races, with larger gains observed in the krill oil group. Overall, it appears that the krill oil group maintained higher choline concentrations than the placebo group, and this effect was most pronounced in the regular-distance triathlon. Repeated measures ANOVA testing the effect of time point (prerace, 0 h post-race, and 1 d post-race), supplement type (krill oil vs. placebo) and race distance (Ironman-distance vs. regular-distance) on choline levels (μmol/L) supports this interpretation of the data: there were significant main effects of time point (*F*_2,86_ = 55.9, *p* < 0.001; $\eta_{\text{p}}^{2}$ = 0.49) and supplement type (*F*_1,43_ = 18.7, *p* < 0.001; $\eta_{\text{p}}^{2}$ = 0.30), as well as significant time point × race distance (*F*_2,86_ = 11.4, *p* < 0.001; $\eta_{\text{p}}^{2}$ = 0.22) and supplement type × race distance (*F*_2,86_ = 9.8, *p* = 0.003; $\eta_{\text{p}}^{2}$ = 0.19) interaction effects. There were no other significant main or interaction effects. *Post-hoc* tests revealed a significant decrease in choline concentration from pre-race to race finish in the Ironman distance athletes (*p* < 0.001) and the regular-distance athletes (*p* = 0.006), with a more pronounced decrease in the Ironman group (*p* < 0.001). Furthermore, the krill oil intervention was associated with overall higher levels of choline in the regular-distance triathlon (*p* = 0.02) and significantly more pronounced increases in choline concentrations from race finish to the day after the race across both distances relative to the placebo group (*p* = 0.039).

### Betaine

**Figure 2:**
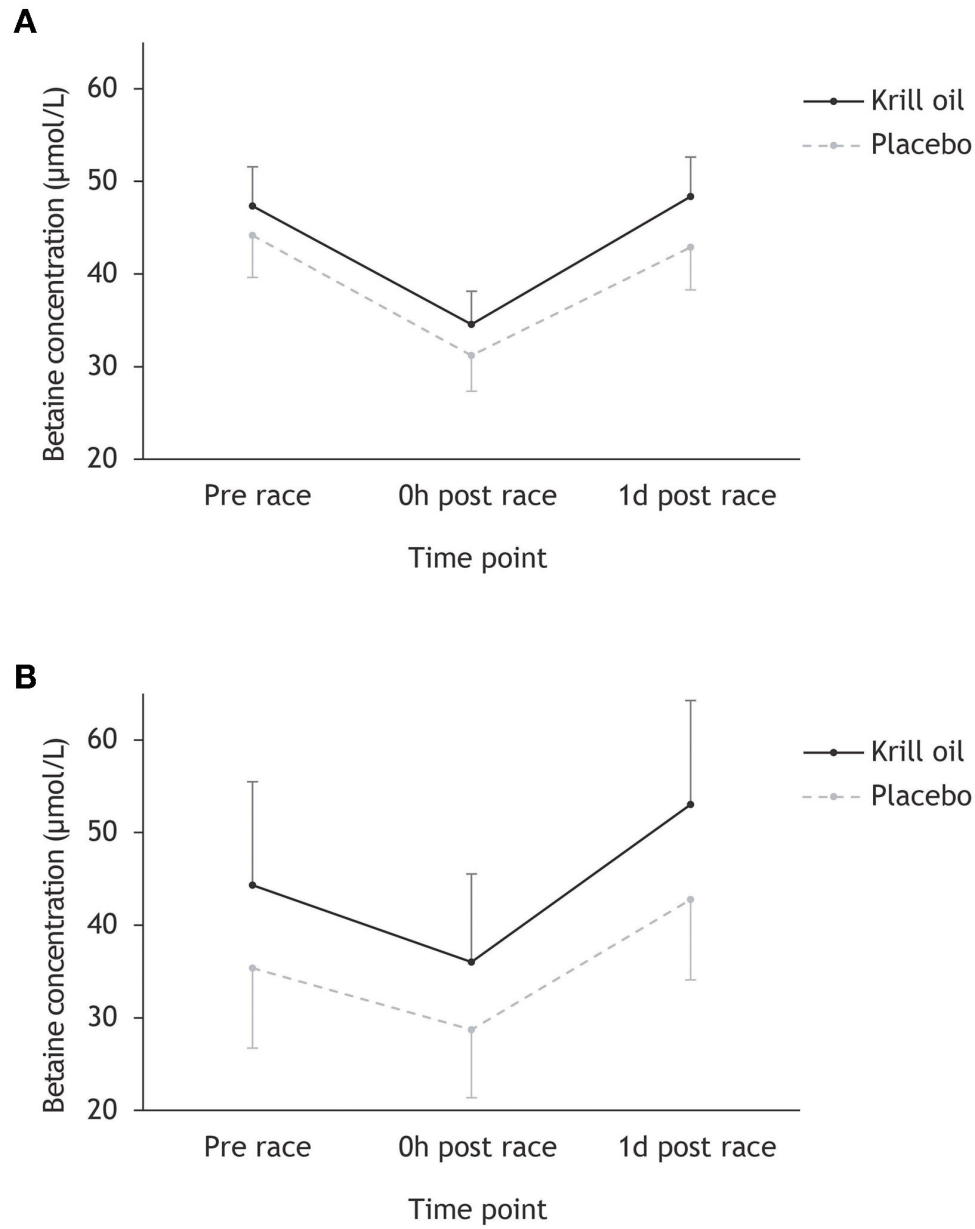
Mean (+/– 95% CI) betaine concentrations (μmol/L) for the krill oil group and the placebo group across the three time points. Results are displayed separately for the Ironman-distance triathlon **(A)** and the regular-distance triathlon **(B)**.

Figure 2 shows that betaine concentrations follow a similar pattern across time points to choline, with participants completing the Ironman-distance experiencing a drop from pre-race to race finish from 45.9 to 33.0 μmol/L and participants competing in the regular-distance triathlon experiencing a drop from 38.7 to 31.5 μmol/L. Recovery in betaine concentrations was observed the following day. Repeated measures ANOVA found a significant main effect of time point (*F*_2,86_ = 36.7, *p* < 0.001; $\eta_{\text{p}}^{2}$ = 0.46), a marginally non-significant effect of supplement type (*F*_1,43_ = 4.03, *p* = 0.051; $\eta_{\text{p}}^{2}$ = 0.09) and no significant time point × race distance (*F*_2,86_ = 2.00, *p* = 0.056; $\eta_{\text{p}}^{2}$ = 0.07) or supplement type × race distance (*F*_2,86_ = 0.57, *p* = 0.453; $\eta_{\text{p}}^{2}$ = 0.01) interaction effects. *Post-hoc* tests revealed a significant decrease in betaine concentrations from pre-race to race finish and a significant increase in betaine concentrations from race finish to the day after the race (*p* < 0.001).

### DMG

**Figure 3:**
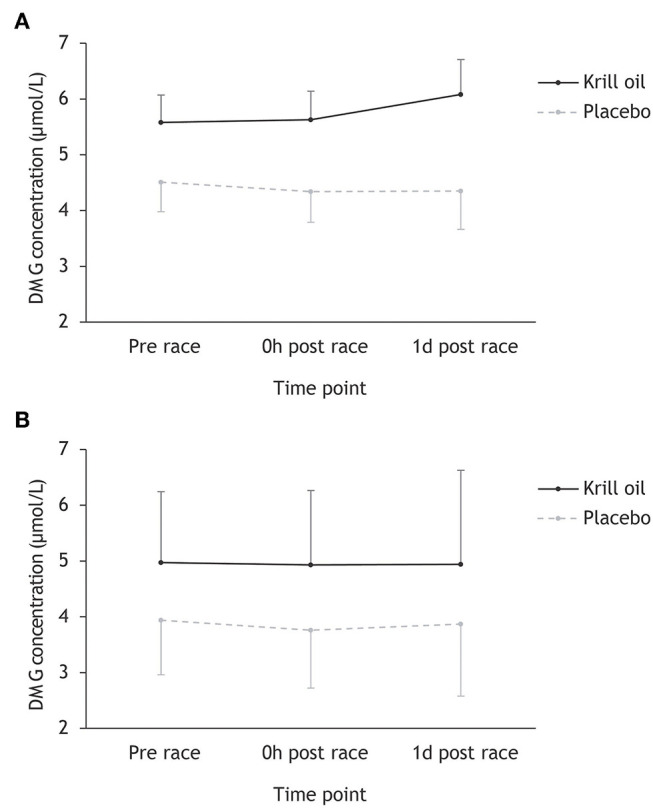
Mean (+/– 95% CI) DMG concentrations (μmol/L) for the krill oil group and the placebo group across the three time points. Results are displayed separately for the Ironman-distance triathlon **(A)** and the regular-distance triathlon **(B)**.

Inspection of Figure 3 reveals that DMG concentrations did not change during or after any of the triathlons, and that the krill oil group displayed higher DMG levels throughout the entire study period. This description of the data was supported by the repeated measures ANOVA, which found a significant main effect of supplement type (*F*_1,43_ = 8.26, *p* = 0.006; $\eta_{\text{p}}^{2}$ = 0.16), but no other significant main or interaction effects.

### Other Choline Metabolites

As can be observed in Table 3, significant main effects of time point were observed for arginine, methionine (decreases from baseline), creatine, cysteine, and homocysteine (increases from baseline) (all *p* < 0.001), but there were no significant effects of supplement type on any of these choline metabolites.

**Table 3:** Serum free choline and choline metabolite concentrations (mean ± SD) for each time point (μmol/L).

**Choline metabolites**	**Group**	**Pre-race**	**0 h post-race**	**1 d post-race**	**$\eta_{\text{p}}^{2}$ (tp)**	**$\eta_{\text{p}}^{2}$ (suppl)**
Choline	Krill oil	16.4 ± 2.8	11.3 ± 1.7	16.2 ± 3.8	0.492^***^	0.303^***^
	Placebo	15.1 ± 3.2	10.3 ± 1.3	13.5 ± 1.9		
Arginine	Krill oil	99 ± 15	71 ± 13	92 ± 17	0.421^***^	0.001
	Placebo	110 ± 27	72 ± 16	88 ± 21		
Betaine	Krill oil	47 ± 12	35 ± 9	49 ± 11	0.460^***^	0.086
	Placebo	42 ± 8	31 ± 7	43 ± 8		
Creatine	Krill oil	83 ± 11	102 ± 17	91 ± 16	0.487^***^	0.005
	Placebo	84 ± 9	101 ± 13	87 ± 12		
Cysteine	Krill oil	273 ± 23	355 ± 43	310 ± 39	0.308^***^	0.001
	Placebo	290 ± 35	342 ± 50	306 ± 35		
DMG	Krill oil	5.5 ± 1.4	5.5 ± 1.4	5.9 ± 1.9	0.004	0.161^**^
	Placebo	4.4 ± 0.7	4.2 ± 0.9	4.2 ± 0.9		
Homocysteine	Krill oil	9.7 ± 1.8	12.6 ± 3.7	14.0 ± 4.2	0.165^***^	0.006
	Placebo	10.5 ± 2.5	11.5 ± 3.2	12.7 ± 3.5		
Methionine	Krill oil	36 ± 11	27 ± 6	46 ± 11	0.236^***^	0.008
	Placebo	38 ± 9	27 ± 8	44 ± 14		
TMAO	Krill oil	5.7 ± 3.3	6.9 ± 7.1	15.4 ± 16.4	0.019	0.018
	Placebo	7.4 ± 8.7	7.5 ± 9.2	7.4 ± 8.7		
TML	Krill oil	0.6 ± 0.2	0.7 ± 0.2	0.9 ± 0.6	0.005	0.020
	Placebo	0.7 ± 0.4	0.7 ± 0.1	0.8 ± 0.4		

$\eta_{p}^{\text{2}}$-values represent effect size for time point (tp) and supplement group (krill oil vs. placebo) in a repeated measures ANOVA, holding race distance constant. Statistically significant effects are marked in bold, where

** denotes p < 0.01 and

*** *denotes p < 0.001*.

## Discussion

As seen in previous sport studies (23), the present investigation confirmed that there is a clear but temporary reduction in circulating, free choline and several of its metabolites in athletes participating in triathlons. Because choline depletion may be linked to a reduction in endurance performance (24, 25), the results highlight the need for increasing choline intake before strenuous endurance exercise.

### At Start of Race

The investigation in triathletes showed that, when circulating choline concentrations of the control group (10.5 μmol/L) before the race are within the range for healthy subjects [7–12 μmol/L, (28, 33, 34)], as seen in participants of the “regular-distance” 2018 Oslo triathlon, then a 5-week krill oil supplementation can significantly increase plasma choline concentrations to 15.6 μmol/L. Krill oil intake leads to an increase in free choline concentrations by providing PC from which the enzyme phosphatidylethanolamine N-methyltransferase synthesizes choline in the liver (16). However, when free choline levels were already high (16.4 μmol/L), as seen in the analysis of the Ironman-distance Norseman 2018 and 2019, then further supplementation with 4 g daily krill oil did not increase serum choline levels. It is unclear why the levels were in the higher range compared to what is usually found in healthy individuals (33), since the diet-related information from the athletes did not provide enough details to be able to accurately calculate choline intake. It is not expected that the high-carbohydrate diet of triathletes substantially influenced choline levels, however, pre-race nutrient “loading” may still have contributed to the unusually high pre-race levels observed in our study. It is for example known that a small breakfast can lead to 25–30% higher choline concentrations in comparison to a fasting condition (35).

The effect of krill oil intake on choline values can be partly hidden by homeostatic regulations, tissue uptake or conversion to metabolites (34, 36). Inasmuch, choline can be irreversibly metabolized to betaine in the liver and kidney, which is a two-step process involving choline dehydrogenase and betaine aldehyde dehydrogenase (37, 38). From betaine, a methyl group can be transferred to homocysteine for the synthesis of methionine, thereby generating DMG (30). Indeed, increased betaine (47 vs. 42 μmol/L in krill oil vs. placebo group) and DMG concentrations (5.5 vs. 4.4 μmol/L in krill oil vs. placebo group) were observed in both race settings with values that are substantially higher to what is usually detected in healthy cohorts (betaine: 30–39 μmol/L and DMG: 2.5–4.0 μmol/L) (33).

From a performance perspective, the increased betaine concentration in the krill oil group might be of importance, since betaine is thought to promote muscle function and plasma volume expansion as shown after betaine supplementation in some studies, albeit not by all (39). Similar results of increased fasting plasma choline (+28.4%, *p* < 0.001), betaine (+26.6%, *p* < 0.001), and DMG (+33.7%, *p* < 0.001) concentrations were described previously after 28 days of 4.5 g of krill oil supplementation in healthy volunteers (40).

### Immediately After the Race

A drop in circulating choline concentrations of up to 40% following prolonged high-intensity exercise has been reported previously, with no effect in shorter or less intense physical activities (19, 21, 24, 25). Our study confirmed that the more intense and longer the activity, the higher reduction in free choline can be observed. Norseman Extreme Triathlon is one of the toughest triathlons in the world (41, 42). It starts with a 3,800-m swimming leg in the Hardanger Fjord in Norway. Then the athletes continue with 180 km by bicycle, which is followed by a 42-km marathon to the top of Mt. Gaustatoppen. The total elevation gain of the race is 5,200 m, and the average race time in our test subjects was in excess of 14 h. In comparison, the test subjects completed the Olympic-distance at Oslo Triathlon in an average of 2 h and 42 min and the sprint distance in around 1 h and 30 min.

It is therefore not surprising that choline levels dropped substantially more during the Norseman triathlon than during the Oslo triathlon. It has been suggested that the decrease in circulating choline during intense sports activities is because of an increased demand for choline as a methyl-group donor (22). If not enough choline is available during race settings, then muscle performance might be negatively affected, since less of the neurotransmitter acetylcholine can be synthesized (20, 43). A substantial drop at the end of the race was also seen in the betaine profile of both cohorts, whereas DMG remained unaffected. This might reflect that betaine can provide methyl groups when choline is in short supply, even though the body cannot directly convert betaine into choline (44).

### One Day After the Race

In the case of the much tougher Norseman, post-race values after 1 day for choline and betaine recovered to higher values in the krill oil compared to the control group, but they did not exceed pre-race values. However, values from the Oslo triathlon recovered in both groups to above pre-race values. An explanation might be that more of these nutrients were utilized during the longer and harder Norseman competition (22). Importantly, compared to the placebo group, there was a greater increase in choline from race finish to the day after the race in the krill oil group.

### Strengths and Limitations

One major strength of this study is that it was conducted in a competition setting with a group of highly motivated triathletes. For many participants, the Norseman Xtreme Triathlon represents one of the highlights of their sporting life, and this means that their preparations in terms of training, diet, rest, and race day planning, as well as their race effort, accurately reflect their athletic potential and real-world behaviors. Furthermore, this approach also suggests that the present study can be used as a practical guide to establish pre-race nutritional strategies for improving levels of choline in active triathletes. However, this approach also means that there is less experimental control. Further examinations on the benefits of krill oil for sports nutrition should aim at a more controlled study set up, where food intake can be closely monitored and an increase in performance and shorter regeneration times can be accurately measured. A dose-response study that correlates the amount of supplemented PC with the outcome measurements would further strengthen the findings of this field study.

### Future Directions

An interesting aspect for a future study could also be the direct comparison of supplementation of equal amounts of choline from PC vs. choline salt for endurance exercise. It has been suggested that 60% of choline in inorganic salts is lost to conversion to trimethylamine (TMA) by intestinal bacteria (45). On the other hand, choline in the form of PC is considerably less converted to TMA as demonstrated in a single-dose study with krill oil (14), potentially resulting in more efficient delivery of choline.

In conclusion, by increasing the availability of choline and its metabolites in the body, supplementation with krill oil might benefit endurance athletes, military personnel or anyone who is exposed to choline-depleting physical activities or has low choline starting levels. If thereby also athletic performance is improved, e.g., by influencing impaired muscle capacity and cognitive function, should be the focus of future studies.

## Data Availability Statement

The raw data supporting the conclusions of this article will be made available by the authors, without undue reservation.

## Ethics Statement

The studies involving human participants were reviewed and approved by Ethical approval was obtained from the Regional Committees for Medical and Health Research Ethics in Norway (Protocol number: REK Sør-Øst 2016/932). The study was performed in accordance with the declaration of Helsinki. The patients/participants provided their written informed consent to participate in this study.

## Author Contributions

AS, LJ, and JH were involved in designing the study and project supervision. JM, CN, AS, and JH was responsible for sample collection and analysis. AS performed the statistical analysis. LB and AS wrote the original draft. LJ, JM, CN, and JH was involved in reviewing and editing. All authors contributed to the article and approved the submitted version.

## Conflict of Interest

AS, LJ, and LB are employees of Aker Biomarine Antarctic AS, Norway that has provided the krill oil and has sponsored the study. The remaining authors declare that the research was conducted in the absence of any commercial or financial relationships that could be construed as a potential conflict of interest.
